# How Does Contrast Administration Influence Creatinine Dynamics in Trauma Patients With Acute Kidney Injury?

**DOI:** 10.7759/cureus.83788

**Published:** 2025-05-09

**Authors:** Nathaniel Grabill, Mena Louis, Mariah Cawthon, Morgan Krause, Bradley Kuhn

**Affiliations:** 1 General Surgery, Northeast Georgia Medical Center Gainesville, Gainesville, USA; 2 Trauma and Acute Care Surgery, Northeast Georgia Medical Center Gainesville, Gainesville, USA

**Keywords:** acute kidney injury, creatinine difference, length of hospital stay (los), major trauma, renal function

## Abstract

Background

Acute kidney injury (AKI) is a serious complication frequently encountered among trauma patients, with incidence rates varying significantly depending on injury severity and diagnostic interventions such as contrast-enhanced imaging. Serum creatinine (Cr) is commonly used to assess renal function; however, its variability following contrast administration and the implications for clinical outcomes in trauma-related AKI remain poorly defined. This study aimed to evaluate the variability of Cr changes from admission to AKI diagnosis following contrast-enhanced CT and their relationship with clinical outcomes such as hospital length of stay (LoS), dialysis requirements, and mortality in trauma patients.

Methods

A retrospective analysis was conducted on adult trauma patients admitted to a Level 1 trauma center who developed AKI after receiving intravenous iodinated contrast during CT scans within 24 hours of admission. Patients with end-stage renal disease (ESRD), advanced chronic kidney disease, pre-admission dialysis, or incomplete baseline Cr data were excluded. Cr changes were calculated from admission to AKI diagnosis. Associations between Cr changes and clinical outcomes, including LoS, dialysis requirements, and mortality, were assessed using statistical methods.

Results

Cr changes showed significant variability, ranging from -1.6 mg/dL to 5.7 mg/dL. However, no statistically significant association was found between contrast-induced Cr changes and hospital LoS, dialysis requirements, or mortality (p > 0.05). These results suggest that Cr fluctuations following contrast administration alone do not reliably predict clinical outcomes.

Conclusion

While Cr changes remain useful for assessing kidney function following trauma and contrast administration, this measure alone has limited predictive value for clinical outcomes in trauma-induced AKI. A more comprehensive approach, incorporating additional clinical factors and novel biomarkers, is necessary for accurate risk stratification and effective management. Future studies should explore integrated assessment tools to improve early detection and personalized management of AKI in trauma patients exposed to contrast-enhanced imaging.

## Introduction

Acute kidney injury (AKI) is a serious complication frequently observed among critically ill trauma patients. AKI involves a sudden decline in renal function, resulting in the accumulation of waste products and disturbances in electrolyte balance [1]. The reported incidence of AKI among trauma patients varies significantly, with studies indicating rates between 10% and 40%, influenced by factors such as injury severity, hemodynamic instability, and the use of nephrotoxic agents like intravenous contrast media during imaging [2]. AKI is associated with increased morbidity and mortality, prolonged hospitalization, and higher risks of chronic kidney disease [3]. Despite advances in trauma care, identifying trauma patients at the highest risk for developing AKI remains challenging, emphasizing the importance of early detection and targeted management strategies [4].

Serum creatinine (Cr) is routinely used as a primary biomarker to diagnose and monitor AKI due to its widespread availability and low cost [5]. Changes in Cr over time, known as creatinine differences (Cr dif), are commonly utilized to gauge the severity and progression of renal impairment [6]. However, Cr is an indirect and relatively late marker of kidney dysfunction; it can also be influenced by non-renal factors such as muscle mass, fluid resuscitation strategies, hydration status, and medications [7]. Thus, Cr alone may not accurately reflect the true degree of renal injury, particularly in trauma patients who receive substantial fluid resuscitation or contrast-enhanced imaging [8]. A clearer understanding of Cr dynamics following contrast administration is therefore critical to improving the assessment and management of AKI in this population.

In trauma settings, rapid identification of AKI risk, especially after contrast exposure, is critical to improving patient outcomes [5]. While serum Cr remains fundamental in assessing renal function, its recognized limitations highlight the need for additional clinical parameters and novel biomarkers that may more accurately predict clinical outcomes [9]. AKI significantly impacts healthcare resource utilization through longer hospital stays, increased medical costs, and elevated requirements for renal replacement therapy (RRT) [10]. Clarifying how intravenous contrast administration specifically influences Cr dynamics could thus help refine risk stratification, enhance personalized patient care, and potentially improve clinical outcomes following trauma-related AKI.

## Materials and methods

Study design

This retrospective cohort study was conducted to evaluate the relationship between changes in serum creatinine (Cr) levels, specifically from hospital admission to the point of AKI diagnosis, and key clinical outcomes in trauma patients who underwent contrast-enhanced CT imaging. The primary objective was to determine whether fluctuations in Cr after contrast exposure were associated with hospital length of stay (LoS), the need for dialysis, or in-hospital mortality. The retrospective design enabled the examination of real-world data across multiple years at a high-volume trauma center.

Study population

The study population included adult trauma patients aged 18 years and older who were admitted to a community-based tertiary referral hospital and Level 1 trauma center between November 2017 and February 2024. Patients were included if they developed AKI during their hospital admission and underwent at least one IV contrast-enhanced CT scan within 24 hours of arrival. AKI was defined according to the Acute Kidney Injury Network (AKIN) criteria, which includes an increase in serum Cr by ≥0.3 mg/dL within 48 hours, or an increase to ≥1.5 times the baseline level, known or presumed to have occurred within the preceding seven days. Patients were excluded if they were younger than 18 years of age; had pre-existing end-stage renal disease (ESRD); were on chronic dialysis; had chronic kidney disease stage 4 or 5; or had incomplete medical records, including missing baseline Cr values. These criteria ensured that the study focused specifically on trauma-related AKI potentially influenced by contrast exposure, while minimizing confounding from pre-existing renal dysfunction.

Data collection

Data were extracted retrospectively from the hospital’s electronic health record system using standardized data abstraction procedures. Collected variables included demographic characteristics such as age and sex, relevant comorbidities including hypertension and diabetes mellitus, trauma mechanism and severity (where available), serial Cr measurements during hospitalization, and contrast administration parameters, including the type, timing, and volume of iodinated contrast delivered during initial imaging. Outcome variables included hospital LoS, requirement for dialysis at the time of discharge, and inpatient survival status. These data elements were selected to provide a comprehensive view of the clinical course from admission through discharge, specifically related to renal function and recovery.

Statistical analysis

Descriptive statistics were used to characterize the study population. Continuous variables, such as creatinine difference (Cr dif) and hospital LoS, were reported as mean with SD or median with IQR, depending on data distribution. Categorical variables, including dialysis status and survival outcome, were presented as frequencies and percentages. For inferential analysis, a two-tailed independent samples t-test was initially performed to compare Cr dif between survivors and non-survivors. Due to unequal variance observed between the groups, Welch’s t-test was employed to confirm the robustness of these comparisons. To evaluate whether Cr dif was associated with LoS, linear regression analysis was conducted, with the coefficient of determination (R²) and p-values reported to assess the strength and significance of the correlation. A one-way ANOVA was used to explore whether Cr dif values differed significantly among four patient groups stratified by dialysis outcome at discharge: no dialysis, short-term dialysis, long-term dialysis, and unknown/uncertain dialysis continuation. Because the residuals of both regression and ANOVA models violated normality assumptions based on Shapiro-Wilk testing (p < 0.0001), non-parametric alternatives, including the Mann-Whitney U test and Kruskal-Wallis test, were also performed to confirm findings. All statistical analyses were performed using MedCalc Statistical Software, version 23.0.2. A p-value of less than 0.05 was considered statistically significant across all analyses. Residual normality was further evaluated using the D’Agostino-Pearson omnibus test, and appropriate statistical adjustments were made when assumptions were not met.

Ethical considerations

The study protocol was reviewed and approved by the Institutional Review Board (IRB) at WCG (Approval #1-1765055-1). All data used in the study were de-identified to ensure the confidentiality of patient information. Given the retrospective nature of the study and the exclusive use of anonymized data, the requirement for informed consent was formally waived by the IRB. The research adhered to all applicable institutional and national guidelines for ethical conduct and data protection.

## Results

A total of 113 trauma patients (Table 1) met the inclusion criteria and were included in the final analysis. The median age of the study population was 55 years (IQR: 45 to 68 years), and 71 patients (62.8%) were male. Among the cohort, 53 patients (47%) had pre-existing hypertension, and 34 patients (30%) had diabetes mellitus, both of which are recognized risk factors for AKI. All patients received at least one IV contrast-enhanced CT scan within 24 hours of admission.

**Table 1:** Baseline characteristics of the study population (n = 113). Patient demographics and key clinical variables, including comorbidities, initial renal function, and creatinine changes from admission to AKI diagnosis. All patients received contrast-enhanced CT imaging within 24 hours of admission. AKI: Acute kidney injury.

Variable	Value
Age, median (IQR)	55 years (45-68)
Male	71 (62.8%)
Hypertension	53 (46.9%)
Diabetes mellitus	34 (30.1%)
Contrast-enhanced CT within 24 hours	113 (100%)
Admission creatinine, mean ± SD	1.85 ± 1.02 mg/dL
Creatinine difference (Cr dif), mean ± SD	1.29 ± 1.85 mg/dL

Creatinine difference and survival

**Figure 1:**
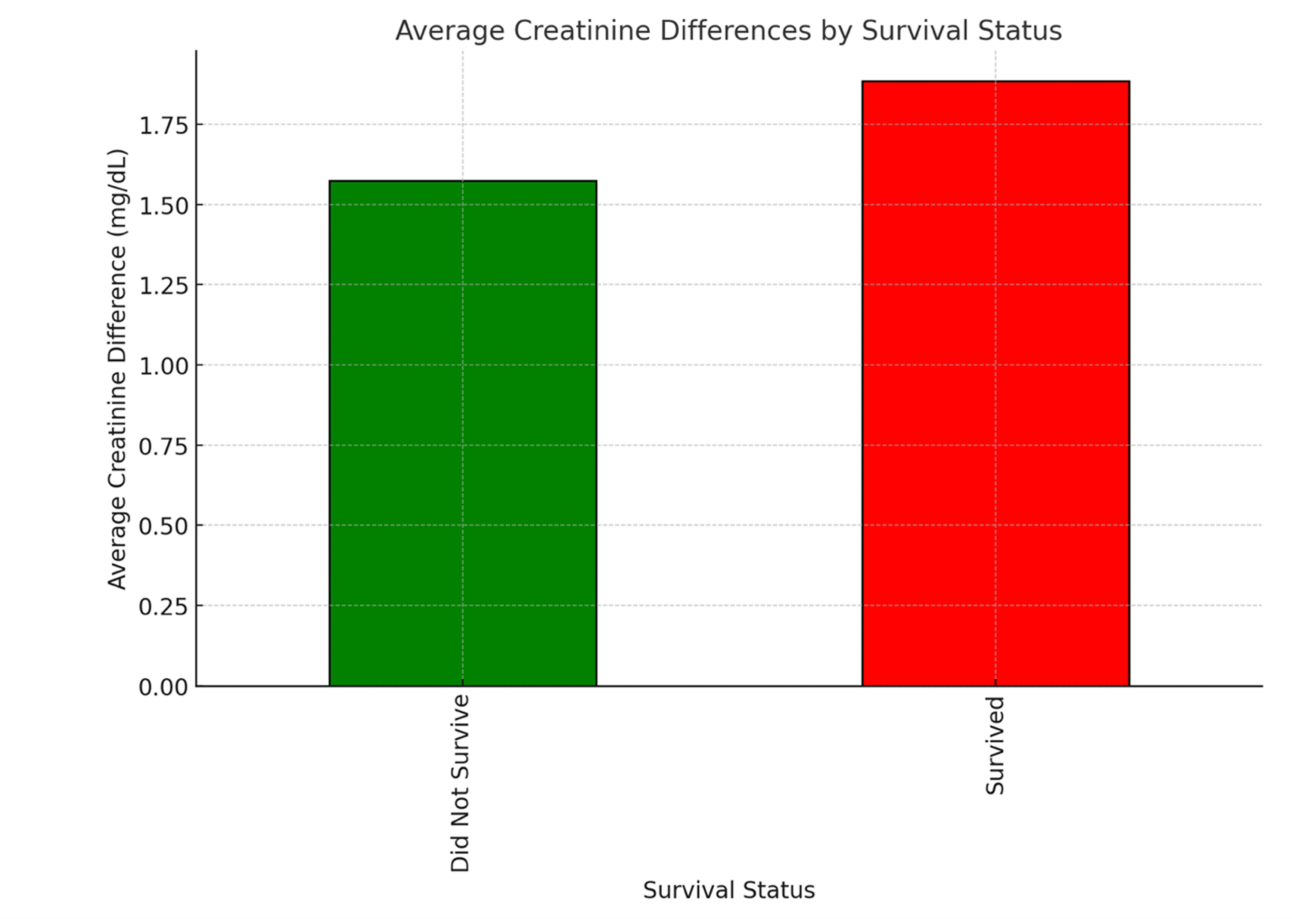
Average creatinine differences by survival status. Shows the average creatinine differences (Cr dif) between survivors and non-survivors among trauma patients with AKI. AKI: Acute kidney injury.

A two-tailed independent samples t-test was used to compare changes in serum Cr (Cr dif) between survivors and non-survivors. Survivors had a mean Cr dif of 1.63 ± 1.13 mg/dL, while non-survivors had a mean Cr dif of 0.94 ± 2.55 mg/dL. Although the difference was not statistically significant (p = 0.09), the trend suggests that greater changes in Cr were observed among survivors (Figure 1). This may indicate that Cr fluctuations, in isolation, are insufficient to predict mortality in this population, and that patient outcomes are more likely influenced by trauma severity, pre-existing comorbidities, and the timing and appropriateness of clinical interventions.

Admission creatinine levels and survival

Admission Cr values were also compared to determine their predictive utility for survival outcomes. The mean admission Cr was 1.82 ± 0.98 mg/dL in survivors and 1.90 ± 1.15 mg/dL in non-survivors, with a p-value of 0.78. These findings indicate no significant difference between the groups, suggesting that baseline renal function on admission does not effectively differentiate survivors from non-survivors in trauma-related AKI. This reinforces the complexity of AKI pathophysiology in trauma, where Cr alone may not capture the full clinical context, especially when fluid shifts, muscle breakdown, and other systemic responses are considered.

Relationship between creatinine difference and LoS

Linear regression analysis was performed to determine whether Cr dif was associated with hospital LoS. The regression model yielded an R² value of 0.020 and a slope coefficient of -0.0091, with a p-value of 0.13. This weak association implies that Cr dif accounted for only 2% of the variation in LoS. These results suggest that changes in Cr during hospitalization do not meaningfully predict the length of inpatient stay, and that other variables, such as injury severity, presence of complications, or intensive care requirements, likely contribute more substantially to LoS.

Analysis of creatinine difference across dialysis outcomes

**Figure 2:**
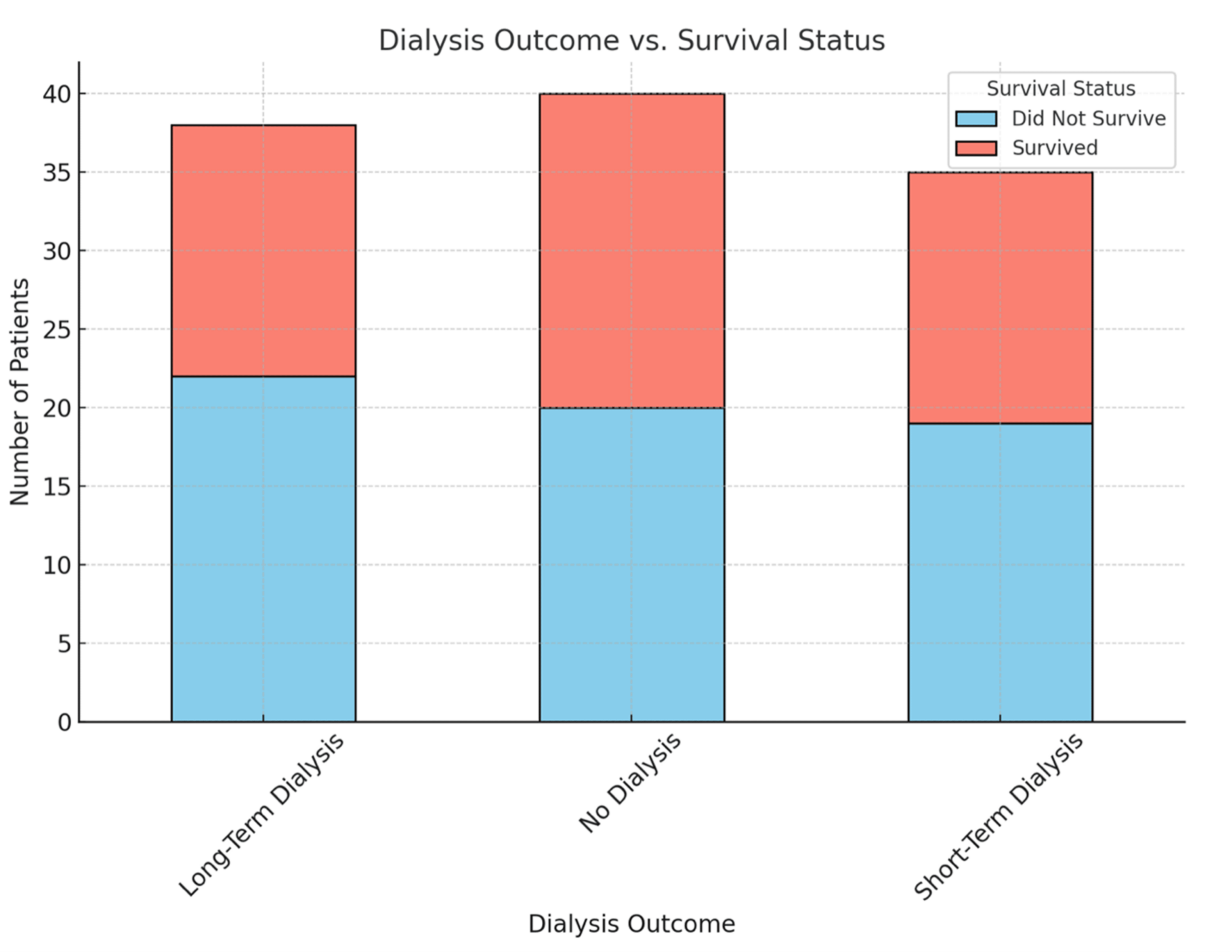
Dialysis outcome vs. survival status. Shows the relationship between dialysis outcomes and survival status among trauma patients with AKI. It highlights the distribution of survival and non-survival rates within each dialysis category, indicating that survival varies across different dialysis requirements. AKI: Acute kidney injury.

To evaluate whether Cr dif values differed by dialysis outcome, a one-way ANOVA was performed across four groups. The mean Cr dif values were 1.72 mg/dL (SD 1.47) for patients not requiring dialysis, 1.72 mg/dL (SD 1.11) for those requiring short-term dialysis, 0.93 mg/dL (SD 2.72) for those requiring long-term dialysis, and 1.46 mg/dL (SD 0.71) for patients with unknown or uncertain dialysis needs (Figure 2). The ANOVA produced an F-ratio of 0.819 with a p-value of 0.49, indicating no significant differences in Cr dif across the groups. These results suggest that changes in Cr levels were not predictive of dialysis need or trajectory, consistent with the understanding that dialysis initiation is influenced by multiple clinical factors beyond Cr dynamics, including electrolyte imbalance, fluid overload, and hemodynamic instability.

Residuals and normality analysis

**Figure 3:**
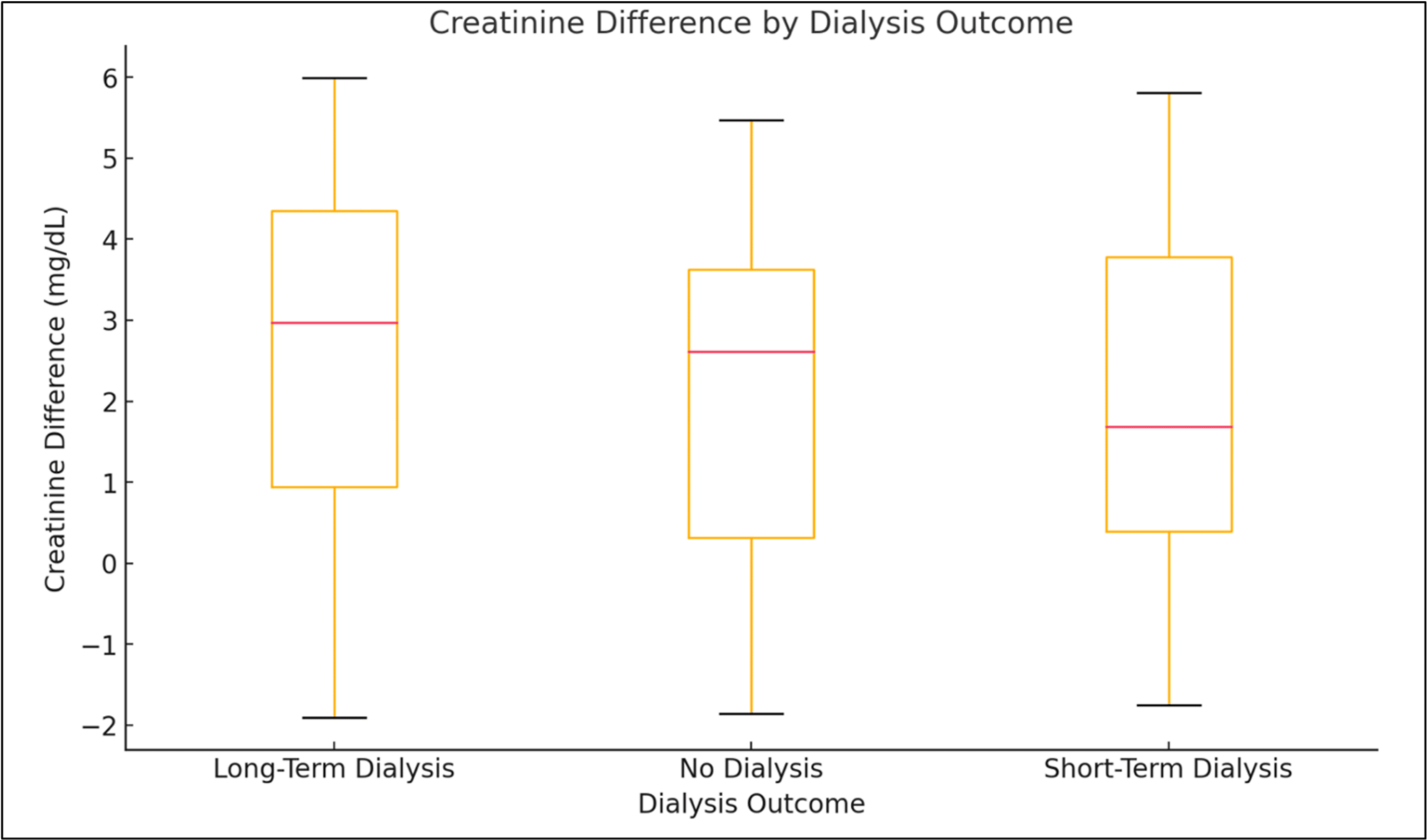
Creatinine difference by dialysis outcome. The ANOVA results indicated that there were no statistically significant differences in creatinine changes between these groups. This suggests that creatinine differences during hospitalization were not a useful indicator of the need for dialysis at discharge.

Residuals from both regression and ANOVA models were assessed for normality using the Shapiro-Wilk test and D’Agostino-Pearson test, both of which indicated significant deviation from normality (p < 0.0001). To validate findings, non-parametric tests were conducted, including the Mann-Whitney U test for survival comparisons and the Kruskal-Wallis test for dialysis outcome groups. These analyses confirmed the lack of statistically significant differences, supporting the robustness of the initial results despite distributional violations (Figure 3).

Summary of findings

**Figure 4:**
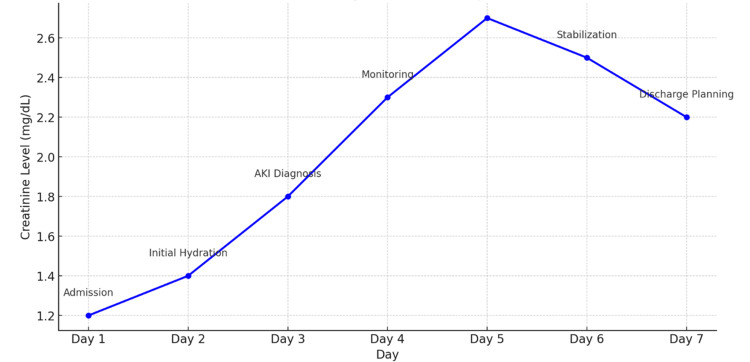
Progression of creatinine levels over time. Line graph showing the progression of creatinine levels over time (in days) during hospitalization. Markers at each time point indicate significant clinical interventions associated with changes in creatinine levels.

In this cohort of trauma patients with AKI following contrast-enhanced imaging, changes in Cr from admission to AKI diagnosis were not significantly different between survivors and non-survivors, though a trend toward greater differences in survivors was noted. Admission Cr levels also failed to predict survival, highlighting the limitations of baseline renal function as a prognostic tool in this setting. Similarly, changes in Cr were not associated with hospital LoS, which appears to be more strongly influenced by trauma-related variables and critical care factors. Lastly, creatinine differences were not predictive of dialysis outcomes, reflecting the multifactorial nature of RRT decision-making in trauma patients. Collectively, these findings suggest that while Cr remains a widely used indicator of kidney function, its fluctuations following contrast administration do not reliably predict clinical outcomes in this population (Figure 4). Broader clinical assessments and the use of alternative or adjunctive biomarkers are likely necessary to improve risk stratification and management in trauma-associated AKI.

## Discussion

This study examined the relationship between changes in serum creatinine levels following contrast-enhanced imaging and clinical outcomes in trauma patients diagnosed with AKI. While AKI is recognized as a serious complication in critically ill patients, particularly those with traumatic injuries, early identification and appropriate intervention remain critical to improving outcomes [11]. Our analysis explored whether variations in creatinine levels from admission to the time of AKI diagnosis, termed Cr dif, were associated with hospital LoS, the need for dialysis, or in-hospital mortality.

The findings showed no statistically significant correlation between Cr dif and hospital LoS. This is consistent with previous studies suggesting that renal function, while important, is only one aspect influencing recovery in trauma patients [12]. The length of hospitalization is often driven by multiple clinical factors, including the extent and nature of traumatic injuries, the development of complications such as infections or sepsis, and the need for prolonged intensive care [13]. In this context, creatinine changes following contrast administration, though useful in assessing renal function, may not provide sufficient predictive insight into a patient's hospitalization course.

Similarly, there was no statistically significant difference in Cr dif between survivors and non-survivors. This observation aligns with existing literature indicating that the presence of AKI alone may not reliably predict mortality, particularly in patients with complex, multisystem trauma [14]. Although AKI is associated with an increased risk of death, the lack of association in our study may reflect the multifactorial nature of trauma outcomes. Variables such as injury severity scores, presence of comorbidities, hemodynamic status, and the timing of supportive interventions may play a more dominant role in determining survival than creatinine kinetics alone. These results suggest that a single parameter like Cr dif, while clinically meaningful, cannot replace a comprehensive risk assessment strategy.

The analysis also demonstrated that Cr dif was not significantly associated with the need for dialysis at discharge. Although creatinine remains a fundamental marker of kidney function, the decision to initiate RRT is influenced by additional factors such as fluid status, electrolyte disturbances, acid-base imbalances, and hemodynamic stability. This finding reinforces the clinical understanding that dialysis initiation is not based solely on creatinine thresholds and that broader clinical judgment is essential in guiding renal support decisions.

These findings support the notion that while creatinine is a valuable biomarker for monitoring renal function, it has limitations when used in isolation. Trauma patients often receive large volumes of IV fluids early in their hospital course, which can dilute serum creatinine levels and potentially obscure evolving renal dysfunction. Additionally, creatinine is a delayed marker of injury and may not rise until significant nephron damage has occurred, limiting its utility for early diagnosis. These limitations are well-recognized and may explain the lack of strong associations observed between Cr dif and major clinical outcomes in our cohort.

Given these constraints, the integration of additional biomarkers may improve risk stratification and enable earlier detection of AKI in trauma patients. Emerging markers such as neutrophil gelatinase-associated lipocalin (NGAL) and kidney injury molecule-1 (KIM-1) have shown promise in detecting renal injury earlier than serum creatinine [15]. Future studies should explore the use of these markers in conjunction with traditional metrics to develop more accurate, multi-parameter models for predicting AKI progression and patient outcomes following contrast exposure.

Several limitations of this study should be noted. The retrospective design introduces inherent constraints in data quality and limits the ability to draw causal inferences. Reliance on electronic health records raises the possibility of incomplete or missing data, particularly for baseline creatinine values in patients without prior laboratory testing. While all patients in the study received contrast-enhanced imaging within 24 hours of admission, we were unable to control for the exact timing and dosage of contrast administration beyond what was documented. Additionally, the relatively small sample size may have limited the statistical power to detect significant associations, particularly in subgroup analyses or among patients with extreme Cr dif values. Future prospective studies involving larger cohorts with well-documented baseline kidney function, granular injury data, and detailed contrast administration protocols are warranted to validate and build upon these findings.

## Conclusions

Creatinine differences following contrast-enhanced imaging were not reliable predictors of clinical outcomes such as LoS, dialysis requirement, or mortality in trauma patients with AKI. While creatinine remains a useful marker of renal function, its limitations underscore the need for a broader, multi-parameter approach to renal assessment. Clinical decision-making should incorporate factors such as urine output, electrolyte trends, and overall patient status, rather than relying solely on creatinine changes. Future research should focus on validating these findings in larger cohorts and exploring the role of emerging biomarkers to improve early detection and management of AKI in trauma settings.
